# Tracking the dynamics of cellular senescence

**DOI:** 10.18632/aging.204670

**Published:** 2023-04-17

**Authors:** Indra Heckenbach, Morten Scheibye-Knudsen

**Affiliations:** 1 Center for Healthy Aging, Department of Cellular and Molecular Medicine University of Copenhagen, Denmark; 2 Buck Institute for Research on Aging, Novato, CA, 94945, USA

**Keywords:** aging, cellular senescence, nuclear morphology, deep learning, quantitative senescence

Cellular senescence, often defined as a state of permanent cell cycle arrest, is a complex and multifaceted process that arises in diverse contexts. First identified as the end point of replicative exhaustion [1], senescence also arises from DNA damage, mitochondrial dysfunction, oxidative stress, sustained mitogenic signaling through oncogenes, proteostatic stress and other. Senescence is under normal physiological conditions involved in wound healing and embryogenesis. Diverse processes trigger multiple mechanisms that converge into cell cycle arrest and a secretory phenotype. Two key pathways may lead to senescence, including the stress-associated p16/Rb pathway and the p53/p21 damage control mechanism. Senescence has been further characterized by its inflammatory secretome (SASP) that serves to signal immune clearance, although it differs by cell type and method of senescence induction. Despite its variable secretome, the SASP may better define senescence since nondividing cells including neurons and cardiomyocytes may exhibit senescent characteristics, despite being frozen at the G0/G1 stage in the cell cycle.

A major issue for the field has been the lack of universal or exclusive markers for senescence, which may hint that senescence is not a single distinct state. Rather than a binary state, senescence appears to be a dynamic process that includes an early cell cycle arrest, a shallow phase with chromatin remodeling and SASP, and a deep phase with chromatin budding and opening of heterochromatin regions [2]. Replicative senescence appears to be a gradual process, where cells begin to develop a transcriptional profile of senescence early, long before cell cycle arrest and presenting markers such as high p21 and SA-beta-gal [3]. Aging cells utilize temporary cell cycle inhibition and accumulate factors associated with senescence, including p16 [4]. Quiescence, a state of non-proliferation that can be induced by contact inhibition or nutrient deprivation *in vitro*, is considered both reversible and distinct from senescence. However, quiescence appears to be a spectrum with a range of proliferative capacity and prolonged exposure to quiescent conditions can lead to senescence [5].

**Figure 1:**
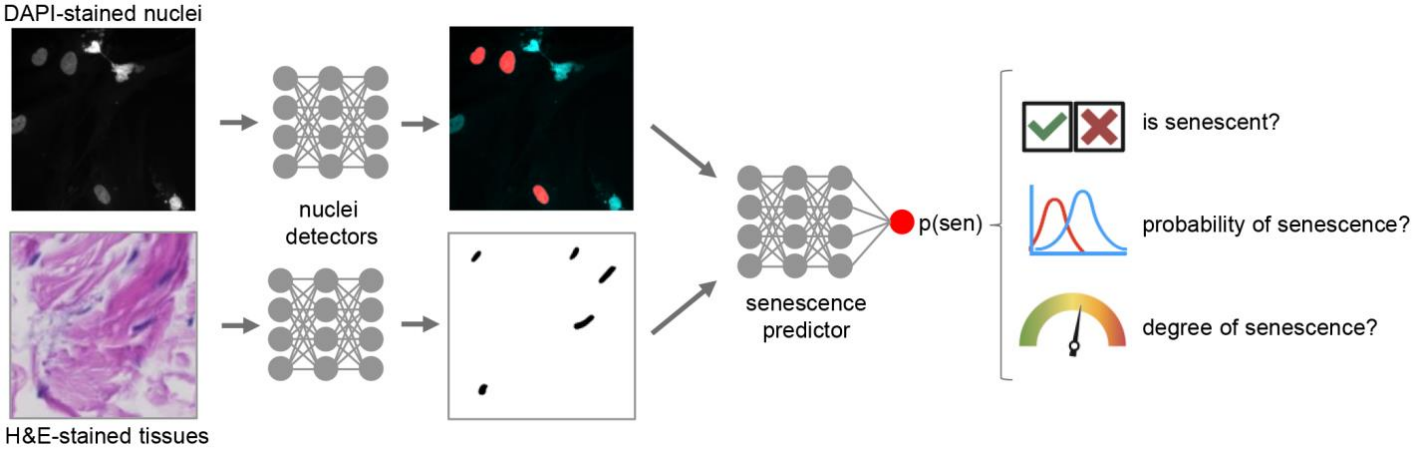
Deep learning for cellular senescence prediction.

We recently introduced deep learning methods to accurately predict senescence from images of nuclear morphology, both in cell cultures and in tissues [6]. This approach uses neural networks to predict senescence for each nucleus identified in a sample, using a softmax output to produce high precision scores (Figure 1). While the higher score values suggest senescence on fibroblasts *in vitro*, the model can be applied to other cell types and nuclei in diverse tissues by comparing numerical scores between conditions. This has been validated in tissue by showing higher mean scores for p21-positive nuclei in human dermis and mouse hepatocytes as well as EdU-negative nuclei, indicating non-proliferation in mouse dermis and testis. The prediction scores, derived from an ensemble of models, can also be interpreted as the probability of senescence. Indeed, we showed that as scores increase there is a higher rate of p21+ nuclei in human dermis and mouse hepatocytes. We also evaluated serum-starved quiescent fibroblasts, finding that at certain times their predicted senescence scores increased above the level of control cells but remained below the level of those induced to senescence by ionizing radiation, indicating the predictor may be sensitive to quiescence or early senescence. The predictor can be used to assess senescence with higher precision, and these findings suggest it may indicate the degree of senescence. More work is needed to understand how deep learned prediction scores represent both types and phases of senescence, but the high-precision quantitative scoring approach appears to be a viable tool to track senescence with greater nuance. This could lead to new insight into the role of senescence in aging and disease.
